# Substrate Utilization by Brown Adipose Tissue: What’s Hot and What’s Not?

**DOI:** 10.3389/fendo.2020.571659

**Published:** 2020-09-25

**Authors:** Ben T. McNeill, Nicholas M. Morton, Roland H. Stimson

**Affiliations:** University/BHF Centre for Cardiovascular Science, University of Edinburgh, Queen’s Medical Research Institute, Edinburgh, United Kingdom

**Keywords:** brown adipose tissue, metabolism, thermogenesis, substrate, obesity, positron emission tomography (PET), lipids, glucose

## Abstract

Our understanding of brown adipose tissue (BAT) function in humans has increased rapidly over the past 10 years. This is predominantly due to the development of powerful non-invasive imaging techniques such as positron emission tomography that can quantify BAT mass and function using metabolic tracers. Activation of BAT during cold–induced thermogenesis is an effective way to dissipate energy to generate heat and requires utilization of multiple energy substrates for optimal function. This has led to interest in the activation of BAT as a potential therapeutic target for type 2 diabetes, dyslipidaemia, and obesity. Here, we provide an overview of the current understanding of BAT substrate utilization in humans and highlight additional mechanisms found in rodents, where BAT more prominently contributes to energy expenditure. During thermogenesis, BAT demonstrates substantially increased glucose uptake which appears to be critical for BAT function. However, glucose is not fully oxidized, with a large proportion converted to lactate. The primary energy substrate for thermogenesis is fatty acids, released from brown adipocyte triglyceride stores. Active BAT also sequesters circulating lipids to sustain optimal thermogenesis. Recent evidence reveals that metabolic intermediates from the tricarboxylic acid cycle and glycolytic pathways also play a critical role in BAT function. Understanding the role of these metabolites in regulating thermogenesis and whole body substrate utilization may elucidate novel strategies for therapeutic BAT activation.

## Introduction

The incidence of obesity, type 2 diabetes mellitus (T2DM) and cardiovascular disease has increased globally over the last 50 years and interventions to curb obesity have been unsuccessful (1). The majority of pharmacological approaches to treat obesity target caloric intake or absorption (2) with substantially less effort targeting the other side of the energy balance equation, energy expenditure. Brown adipose tissue (BAT), a thermogenic organ with substantially higher mitochondrial content than white adipose tissue (WAT) (3) that causes its brown appearance, presents a unique opportunity to address this imbalance as BAT activation increases energy expenditure through oxidation of energy substrates (4).

While well characterised in rodents, until recently BAT was thought only to be found in human infants, however BAT has been identified in the supraclavicular, axillary, and paraspinal regions of adult humans and retains its thermogenic function (5). BAT is stimulated by cold exposure which induces sympathetic activation of β3-adrenergic receptors [β3AR, and possibly β1AR and β2AR (6, 7)] which activates the inner mitochondrial membrane (IMM) transporter uncoupling protein 1 (UCP1) (8, 9). UCP1 transports protons generated from the electron transport chain across the IMM in a process uncoupled from ATP synthesis, generating heat for non-shivering thermogenesis (10). Thermogenesis is an energy intensive process, therefore BAT has a high metabolic demand. A variety of metabolic substrates are utilized by, and are critical for, BAT to initiate and maintain thermogenesis including intracellular triglycerides, circulating free fatty acids (FFA) and glucose. Uptake and utilization of these substrates can improve metabolic health (11).

Recent work has highlighted that BAT thermogenesis utilizes a more complex substrate range than merely glucose and fatty acids (12, 13). This complexity raises important questions regarding the mechanisms regulating BAT activation. In order to target BAT therapeutically, it is essential to understand the key pathways involved in thermogenesis. In this mini-review, we will discuss our understanding of the role and importance of substrate utilization by human BAT and highlight the key remaining questions.

## The Role of Glucose Uptake by BAT

To date, ^18^Fluorodeoxyglucose (^18^FDG)-PET/CT is the most widely used technique to quantify BAT mass and activity in humans by measuring glucose uptake in active BAT (14). This technique has identified multiple BAT depots including the supraclavicular and paracervical regions in adults and revealed that total BAT mass varies widely between subjects from ~10–300 grams (5, 15–17). However, this may be an underestimate as only active depots with significant glucose uptake are detected by this method. Glucose uptake by BAT during warm/thermoneutral conditions ranges from ~10 nmol/g/min using ^18^FDG-PET (5, 18, 19) up to ~50 nmol/g/min using microdialysis (12). Both techniques demonstrate increased glucose uptake during cold stimulation of ~50–200 nmol/g/min in young healthy subjects (5, 12, 18, 19) (**Table 1**), in-keeping with an important role for glucose during thermogenesis. During cold exposure, glucose uptake by BAT is greater than skeletal muscle per gram of tissue but due to the low mass in adults BAT accounts for <1% of total body glucose uptake during thermogenesis, compared with ~50% by skeletal muscle (23). Repeated cold exposure can further increase glucose uptake by BAT (**Table 1**) and increase BAT oxidative metabolism and cold-induced thermogenesis (15, 19). This may be due to increased BAT mass or activation of previously dormant BAT (27, 28). Glucose uptake by BAT correlates with cold-induced thermogenesis in healthy humans, consistent with an important role for glucose during BAT thermogenesis (12, 15). However, this is not observed in all studies and comparisons are often confounded by substantial differences in cooling protocols between studies (**Table 1**) and the often unmeasured contribution of skeletal muscle shivering which accounts for the majority of cold-induced thermogenesis (25, 29).

**Table 1:** Quantification of substrate utilization by human BAT.

Substrate	Lean/overweight adults during warm (nmol/g/min)	Lean/overweight adults during cold (nmol/g/min)	Overweight/obese adults during warm (nmol/g/min)	Overweight/obese adults during cold (nmol/g/min)	Adults with T2DM during cold (nmol/g/min)
**Glucose uptake**	50 (12) – 3h at 24-25°C, microdialysis 10 (20) – duration/temp not specified 10 (5) – 2h, temp not specified 10 (21) – duration/temp not specified	Acute cold exposure studies 110 (18) – 3h at 18°C using water cooled suit 55 (22) – 2h at 18°C using water cooled suit 70 (20) – 2h at 17°C 120 (5) – 2h at 17-19°C 90 (21) – 2h at 17°C 70 (23) – 3h at 18°C using water cooled suit 170 (19) – 2h at 18°C using water cooled suit 160 (12) – 3h at 17°C, microdialysis 69 (15) – ½h, individualised cooling protocol using water cooled suit 45 (24) – 4h at 18°C using water cooled suit **Mean uptake from all studies: 96** Cold acclimation studies 170➔210 (19) – before/after using water cooled suit at 10°C for 2h/day for 5 days a week for 4 weeks 69➔76 (15) – before/after 15-16°C for 2-6h/day for 10 days 45➔50 (24) – before/after individualised cooling protocol using water cooled suit for 2h/day for 5 days a week for 4 weeks	11 (20) – 2h at 23°C	35 (20) – 2h at 17°C	15 (18) – 3h at 18°C using water cooled suit
**FFA uptake**	8 (25) – 22°C, duration not specified 6.5 (26) – 22°C, duration not specified	12 (18) – 3h at 18°C using water cooled suit 13 (22) – 2h at 18°C using water cooled suit 13 (25) – 2h at 6°C using water cooled blanket 12 (26) – 2h at 6°C using water cooled blanket			12 (18) – 3h at 18°C using water cooled suit
**Dietary FA uptake**		3 (24) – >2hrs at 18°C using water cooled suit			
**Glutamate uptake**	25 (12) – 3h at 24-25°C, microdialysis	35 (12) – 3h at 17°C, microdialysis			

Data are approximate mean or median values from the various reported studies to quantify substrate utilization by BAT. The data presented were all obtained using dynamic PET/CT apart from Weir et al. (12) that used microdialysis. The times in hours indicate the duration of warm/cold exposure prior to scanning, or total exposure for (12). Unless otherwise stated, the temperatures indicate the ambient temperature of the room in which the volunteers were housed.

Early studies using static PET scanning demonstrated decreased ^18^FDG uptake by BAT in those with increasing age, weight and fasting glucose, suggesting dysregulation of BAT activity in metabolic disease (28, 30–32). Dynamic PET studies have subsequently quantified the reduction in cold-induced glucose uptake by BAT in obesity and diabetes (**Table 1**). Interestingly, whilst ^18^FDG glucose uptake is reduced in T2DM and age-matched controls compared to a healthy young group (mean age 59 vs 24 years), FFA uptake and BAT oxidative metabolism (measured by ^11^C-acetate) were similar between groups (18). These data indicate that reduced glucose uptake by BAT may not accurately reflect BAT thermogenesis at least in older or diabetic subjects. For example, reduced glucose uptake could be caused by increased insulin resistance without necessarily reducing BAT thermogenesis, potentially calling into question the importance of BAT glucose metabolism (18).

However, other evidence suggests glucose is important for optimal BAT function. The glucose transporters GLUT1 and GLUT4 are expressed in human BAT and are responsible for glucose uptake into brown adipocytes, where it undergoes glycolysis (21, 33). *GLUT4* but not *GLUT1* expression substantially increases during human brown adipocyte differentiation, while inhibition of GLUT4 reduces glucose uptake and thermogenesis, suggesting *GLUT4* is the more important transporter in human BAT (34). In addition, glucocorticoids increase ^18^FDG uptake by human BAT *in vivo* and acutely increase GLUT4 but not GLUT1 mRNA levels in primary human brown adipocytes (33), while prolonged cold exposure increased GLUT4 expression and glucose uptake by BAT *in vivo* in rats (35). In mice, either shRNA knockdown of GLUT1 in interscapular BAT or inhibition of glycolysis *via* the injection of 2-deoxy-D-glucose inhibited *in vivo* optogenetic stimulation of sympathetic nerve activation of non-shivering thermogenesis (36). Finally, suppression of BAT thermogenesis by inhibiting lipolysis also substantially decreases glucose uptake by BAT (23). Overall these data suggest glucose uptake and utilization is important for optimal BAT function, although further work is required to determine the specific role of glucose at thermoneutrality and during thermogenesis in health and disease.

### Glucose Utilization by BAT

**Figure 1:**
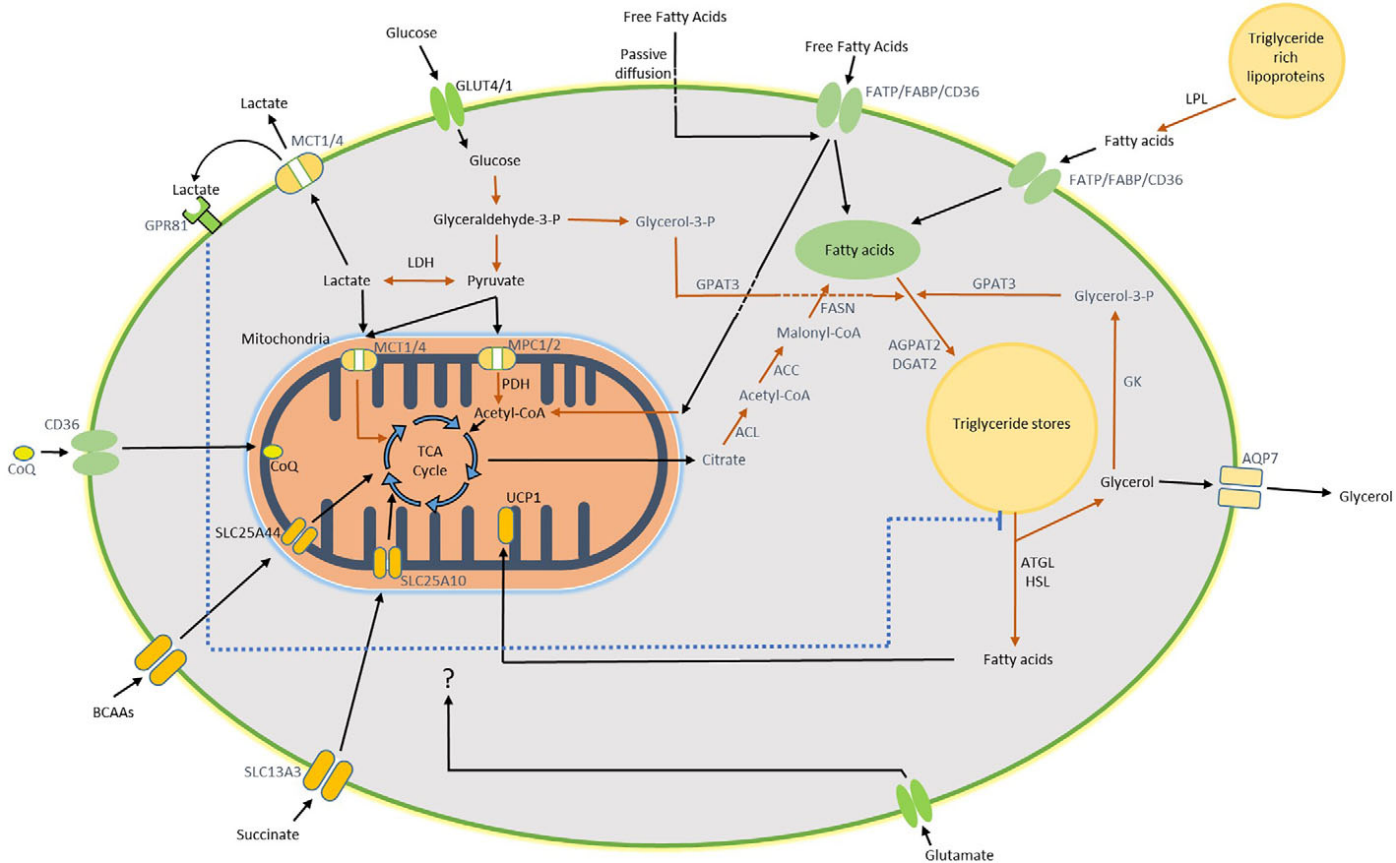
Diagram of substrate utilization and suspected pathways in BAT. Glucose enters the brown adipocyte *via* GLUT4 and GLUT1 where it undergoes glycolysis to form pyruvate. Pyruvate is converted to lactate and exported from the cell by the monocarboxylate (MCT) transporters, this pathway accounts for the majority of glucose uptake by human BAT during both warm and cold exposure. Following export, lactate may activate GPR81 which inhibits lipolysis. Alternatively, pyruvate can enter the mitochondria and be incorporated into the TCA cycle following conversion to acetyl-CoA. Citrate, one of the TCA cycle intermediates, may also be converted to acetyl-CoA in the cytosol as the first step of *de novo* lipogenesis (DNL). A proportion of glucose may be converted to glycerol-3-phosphate (Glycerol-3-P), generated from glyceraldehyde-3-phosphate during glycolysis, to form the backbone for replenishment of intracellular triglycerides (TRGs). Free fatty acids (FFAs) hydrolysed from local TRGs are the primary energy substrate used for uncoupled respiration during thermogenesis, which is mediated by mitochondrial UCP1. Glycerol released by this process can either be exported or recycled through conversion to Glycerol-3-P by glycerol kinase (GK) for subsequent TRG synthesis. In addition to DNL, uptake of circulating FFAs either directly or following lipoprotein lipase (LPL)-mediated hydrolysis of triglyceride rich lipoproteins (TRLs) occurs *via* the fatty acid transporters or potentially by passive diffusion. BAT utilizes other circulating substrates during thermogenesis such as BCAAs, succinate and glutamate which are all likely incorporated into the TCA cycle. Arrows in black indicate substrate transport/movement. Arrows in red represent enzymatic conversion. Wording in black indicate pathway confirmed both in human and rodent BAT. Wording in blue indicate pathways confirmed in rodent BAT. Some reactions have been simplified/omitted for brevity. ACC, acetyl-CoA carboxylase; ACL, ATP citrate lyase; AGPAT2, 1-acyl-sn-glycerol-3-phosphate acyltransferase beta; AQP7, aquaporin-7; ATGL, adipose triglyceride lipase; CD36, fatty acid translocase; CoQ, coenzyme Q; DGAT2, diacylglycerol acyltransferase 2; FABP, fatty acid binding protein; FASN, fatty acid synthase; FATP, fatty acid transport protein; GPAT3, glycerol-3-phosphate acyltransferase 3; GPR81, G-protein coupled receptor 81; HSL, hormone sensitive lipase; LDH, lactate dehydrogenase; MPC1/2, mitochondrial pyruvate carrier 1 and 2; PDH, pyruvate dehydrogenase; SLC13A3, solute carrier family 13 member 3; SLC25A10, solute carrier family 25 member 10; SLC25A44, solute carrier family 25 member 44.

The fate of glucose during thermogenesis and thermoneutral conditions is an area of great interest and uncertainty in human BAT. Glucose intermediates, generated *via* the glycolytic pathway, are likely integral to BAT function (**Figure 1**). Pyruvate, the product of glycolysis, can be converted to acetyl coenzyme A (acetyl-CoA) by pyruvate dehydrogenase (PDH) to feed into the tricarboxylic acid (TCA) cycle. The mitochondrial pyruvate transporters MPC1 and MPC2 are highly expressed in rodent BAT and upregulated by cold exposure (37, 38). Furthermore, we found higher expression of the PDH subunit PDHA1 (and a trend for PDHB) in human BAT compared with WAT, in-keeping with conversion of pyruvate to acetyl-CoA in human BAT (12). Acute β3AR stimulation also increases PDH activity in immortalised murine brown adipocytes (39). While acetyl-CoA may drive oxidative phosphorylation in the TCA cycle to meet increased respiratory demands (39), citrate formed during this process can also be used for *de novo* fatty acid (FA) synthesis (**Figure 1**) (37). FAs formed *via* this pathway are not directly used as fuel for UCP1*-*mediated thermogenesis but instead are used to replenish intracellular triglyceride stores to provide FAs during thermogenesis (40).

Glucose may also contribute to the replenishment of triglyceride stores in BAT through conversion of glyceraldehyde-3-phosphate, one of the intermediates in the glycolytic pathway, to glycerol-3-phosphate which subsequently forms the glycerol backbone during triglyceride synthesis (**Figure 1**). ^13^C-glucose tracing in B3AR-stimulated immortalised murine brown adipocytes revealed that a proportion of glycerol in triglycerides is derived from this process, although most of the glucose was oxidized by these cells (39). BAT expresses pyruvate carboxylase and it is also possible that glucose is converted to oxaloacetate for anaplerosis (41). The contribution of glucose metabolism to these processes in human BAT is currently unknown and requires further investigation. However, we recently identified that conversion of pyruvate to lactate is an important pathway in human BAT (12).

In healthy subjects, lactate release by BAT is ~4-fold greater than WAT and accounts for the majority of glucose uptake at thermoneutrality and >50% during cold activation (12). Similarly, noradrenaline stimulation induces substantial lactate release from rat BAT (42). The lactate transporters monocarboxylate transporter 1 and 4 (MCT1/4) are highly expressed in rodent BAT whilst MCT1 expression is increased by cold exposure or by activation of the sympathetic nerves innervating BAT (36, 43). Pharmacological inhibition of MCT1 (expressed on plasma and mitochondrial membranes) or lactate dehydrogenase (LDH) reduces BAT thermogenesis (36). These data highlight the importance of lactate within BAT, although how lactate regulates thermogenesis is currently unclear. Although the majority of lactate is exported from the cell, a proportion may directly feed into the TCA cycle (44) or be converted to fatty acids (45) (**Figure 1**). The high rates of lactate production in active BAT may also be critical to maintain optimal cellular redox balance during thermogenesis (46, 47). Further, human BAT has high expression of the lactate receptor GPR81 which inhibits lipolysis when activated (12, 48). The high lactate levels in human BAT observed during thermoneutrality may inhibit lipolysis though GPR81 to maximise triglyceride storage for subsequent cold-induced BAT activation (12). Further research is required to investigate the role of lactate in human BAT.

The mechanistic insights into the role of glucose in BAT are predominantly derived from rodent studies and it will be important to confirm these in humans. Understanding the specific roles of glucose metabolism during thermogenesis may identify novel pathways for therapeutic manipulation to activate BAT which may have important implications for improving glucose homeostasis in metabolic disease (49).

## Use of Intracellular Triglycerides

Whilst the fate of glucose is still being elucidated, the role of intracellular triglycerides in BAT is more clearly understood. In rodents, FAs hydrolysed from intracellular triglyceride stores are the primary substrate for thermogenesis (50, 51). Inhibition of lipolysis *in vivo* and *in vitro* substantially inhibits rodent BAT activity (50, 51). Similarly in humans, BAT activation depletes intracellular triglyceride stores, as demonstrated either by the increased radio-density of BAT observed when using CT scanning during cold exposure (19, 22) or by a reduced fat fraction when using MRI (52, 53). Older people, irrespective of T2DM status, have a higher fat fraction in BAT compared with young controls at room temperature and during cold exposure (18). In addition, an increased BAT fat fraction is associated with increased insulin resistance, consistent with a role for BAT activity in metabolic disease (54). However, fat fraction is not substantially reduced in those with greater BAT activity and fat fraction can decrease during cold activation even in subjects without detectable ^18^FDG uptake by BAT (18, 52, 53). One possible reason why fat fraction may not be substantially different between BAT and WAT is the heterogeneous nature of BAT, as human BAT contains regions with higher and lower lipid content (55). In addition, the fat fraction of distinct areas within BAT can increase during thermogenesis, highlighting the importance of triglyceride regeneration to maintain energy stores (55).

Recent evidence in humans strongly suggest that intracellular triglycerides are the primary fuel source for non-shivering thermogenesis as in rodents (**Figure 1**), as inhibition of lipolysis reduces BAT thermogenesis and the change in BAT radio-density during cold exposure (23). Furthermore, quantification of BAT lipolysis was undertaken by measuring glycerol release from BAT using microdialysis in healthy lean men, this was substantially increased during cold exposure (12). FFA release during BAT activation was estimated to be ~65 nmol/g/min which would account for the majority of BAT thermogenesis (12). While export of glycerol occurs most likely *via* the aquaporin 7 glycerol transporter (56, 57), the enzyme glycerol kinase is highly expressed in rodent (50) and human BAT (12, 58), which converts glycerol to glycerol-3-phosphate for subsequent replenishment of triglyceride stores. This strongly suggests that not all glycerol is released so this estimate of local lipolysis by BAT may be a significant underestimate. In addition, glycerol kinase expression is substantially increased by cold exposure in rats (50), highlighting the importance of this pathway during thermogenesis.

## Fatty Acid Uptake by BAT

Although BAT lipid content acutely decreases following cold activation this does not further change during prolonged cold exposure, suggesting that intracellular triglycerides are constantly replenished (**Figure 1**) (22, 59). As discussed above, glucose may contribute to this either through glyceroneogenesis and/or *de novo* lipogenesis. Circulating triglyceride rich lipoproteins (TRLs) and FFAs are also key sources of FAs for this process. Cold exposure increases whole body lipolysis, which enhances circulating fatty acid substrate availability (11, 12). Use of the FA PET tracer ^18^fluoro-6-thia-heptadecanoic acid (^18^FTHA) confirmed uptake of FFAs by BAT during cold exposure (22). FA uptake by BAT during both warm and cold exposure correlates with BAT thermogenesis, in-keeping with an important role for FA uptake in BAT function (25). FFA uptake rates by BAT during cold exposure are ~12.5 nmol/g/min (22) and potentially unchanged in older or diabetic subjects (**Table 1**) (18). FFA uptake by BAT only accounted for ~0.25% of plasma FFA turnover, suggesting that BAT may not have an important role in systemic FFA clearance. FFA uptake by BAT is mediated by several transporters including fatty acid binding protein, fatty acid transport protein, and CD36 (**Figure 1**) (60–62). CD36 also plays an important role in Coenzyme Q uptake and fatty acid oxidation (63). However, the fate of these FFAs following uptake by BAT is currently unclear and other fatty acid tracers are required to determine whether FAs taken up by BAT are oxidized or incorporated into triglyceride stores.

Uptake of FAs from circulating TRLs occurs through the action of lipoprotein lipase (LPL) (64). LPL-mediated TRL uptake by BAT can account for ~50% of systemic TRL clearance in rodents during cold exposure (61, 65, 66). In addition, LPL expression is increased in human BAT but not WAT following cold exposure, highlighting a potentially important role in triglyceride clearance (11). While human *in vivo* data are limited, these suggest this mechanism is present in human BAT. There was detectable uptake of the fatty acid tracer ^18^FTHA by BAT, given orally as part of a lipid meal during cold exposure, confirming that BAT sequesters dietary FAs in humans (24). However, ^18^FTHA by BAT uptake was not increased following a 4-week cold acclimation protocol despite substantially increasing BAT activity. BATs contribution to total dietary fatty acid clearance was estimated to be ~0.3%, although the ^18^FTHA uptake by BAT was proportionately higher per gram of tissue than skeletal muscle or WAT (24). The difference in systemic clearance between species may be due to the lower proportional BAT mass in humans. Prolonged BAT activation using the β3AR agonist mirabegron for 4 weeks in humans increases HDL-C concentrations, potentially as a result of enhanced TRL clearance (49). However, the specific role of BAT in this process is unclear and further research is required to quantify the contribution of human BAT to triglyceride clearance during thermoneutral and cold conditions and whether these FAs are immediately oxidized or used to replenish triglyceride stores. Nevertheless, chronic activation of BAT in humans may induce a more favourable lipid profile which may reduce cardiovascular risk.

## Utilization of Additional Substrates by BAT

While glucose, internal triglyceride stores and FFA are clearly defined as substrates with key roles in BAT thermogenesis, there is emerging evidence that other substrates contribute to BAT function. Using the microdialysis technique, we discovered substantial glutamate uptake by BAT *in vivo* which was increased during cold exposure [∼35 nmol/g/min in the cold vs ∼25 nmol/g/min in warm conditions (**Table 1**) (12)]. Cold exposure did not increase glutamate uptake by WAT, highlighting a specific role for the amino acid during thermogenesis. The function of glutamate in BAT is unclear and requires further study, however glutamate dehydrogenase is expressed in human BAT which converts glutamate to α-ketoglutarate (12), suggesting the glutamate may be oxidized or contributes to anaplerosis. However, glutamate uptake is considerably lower than glucose, indicating this is unlikely to be a major substrate for thermogenesis. In addition, enhanced utilization and depletion of intracellular glutamate may increase glucose uptake by BAT, as glutamate inhibits glucose uptake in white adipocytes (67).

Branched chain amino acids (BCAAs) also contribute to BAT non-shivering thermogenesis. In humans, cold exposure enhances systemic clearance of the BCAAs valine and leucine only in subjects with significant BAT activity (13). Furthermore, expression of the mitochondrial transporter SLC25A44 (which can transport valine and leucine) was higher in human BAT following cold exposure. Highlighting a potential thermogenic role, these BCAAs can be incorporated into TCA cycle intermediates such as succinate, while valine enhanced mitochondrial respiration in stimulated human brown adipocytes *in vitro* (13). Finally, BAT-specific knockdown of *SLC25A4* in mice impaired thermogenesis, highlighting a crucial role for BCAA uptake by BAT *in vivo* at least in mice (13). However, the importance and contribution of these BCAAs to human BAT thermogenesis *in vivo* is unclear at present. Whether the reduced BAT activity associated with obesity contributes to the elevated circulating BCAA levels seen in these subjects (68) is also unknown.

Recent work also implicates the TCA cycle metabolite succinate as a key regulator of BAT thermogenesis. In mice, cold exposure induces sequestration of succinate from the circulation and accumulation in BAT (69, 70). Noradrenaline stimulation of human brown adipocytes *in vitro* also increases intracellular succinate levels (13). Highlighting an important role for this intermediate, succinate treatment increased oxygen consumption in human and murine brown adipocytes *in vitro* and in mice dietary supplementation of succinate enhanced BAT thermogenesis *in vivo*, which is mediated through incorporation of succinate into the TCA cycle (69). Interestingly and as noted above with the BCAAs, circulating succinate levels are higher in obese and diabetic subjects (71) which suggests that succinate supplementation is unlikely to enhance BAT thermogenesis in this group, although this is yet to be tested.

## Conclusion

Our knowledge of substrate utilization by human BAT has improved over the past 10 years, thanks primarily to the use of PET imaging and more recently the application of adipose tissue microdialysis. However, a number of fundamental questions remain such as the fate of glucose and how the triglyceride pool is replenished. While local triglycerides are the primary energy substrate in BAT thermogenesis, it is clear that a number of other substrates play significant roles, although their specific contributions and function remain to be elucidated. If BAT is to be used as a novel therapy for metabolic disease a thorough understanding of the pathways that contribute to BAT thermogenesis is paramount, in addition to determining the maximal metabolic capacity of this organ. In order to achieve sustained improvements in glucose and lipid homeostasis through BAT activation, these pathways may hold the key.

## Author Contributions

BM and RS wrote the manuscript. NM critically revised the manuscript. All authors approved the submitted version.

## Funding

This work was supported by the Medical Research Council (MR/K010271/1, MR/S035761/1), the Chief Scientist Office (SCAF/17/02), and the British Heart Foundation.

## Conflict of Interest

The authors declare that the research was conducted in the absence of any commercial or financial relationships that could be construed as a potential conflict of interest.
